# Magnetically Actuated Continuum Robots: From Engineering to Clinical Translation

**DOI:** 10.34133/cbsystems.0745

**Published:** 2026-11-03

**Authors:** Zhengxin Yang, Yang Jiao, Meng Niu, Yaoyao Cui, Lidong Yang, Qingsong Xu, Li Zhang

**Affiliations:** ^1^ Suzhou Institute of Biomedical Engineering and Technology, Chinese Academy of Sciences, Suzhou 215163, China.; ^2^ Department of Radiology, First Hospital of China Medical University, Shenyang 110001, China.; ^3^ State Key Laboratory of Ultra- precision Machining Technology, Department of Industrial and Systems Engineering, The Hong Kong Polytechnic University, Hong Kong 999077, China.; ^4^ Department of Electromechanical Engineering, Faculty of Science and Technology, University of Macau, Macau 999078, China.; ^5^ Department of Mechanical and Automation Engineering, The Chinese University of Hong Kong, Hong Kong 999077, China.; ^6^ Chow Yuk Ho Technology Centre for Innovative Medicine, The Chinese University of Hong Kong, Hong Kong 999077, China.; ^7^ Department of Surgery, The Chinese University of Hong Kong, Hong Kong 999077, China.

## Abstract

Magnetically actuated continuum robots (MACRs) have emerged as a promising approach for minimally invasive surgery. By using external magnetic fields to apply torques and forces at the instrument tip, MACRs move actuation outside the patient, thus reducing the mechanical complexity of the robot. This architecture enables miniaturization, improved compliance, and improved steering capabilities, especially for navigating intricate intravascular or endoluminal pathways. Despite substantial progress in functional materials, robotic archetypes, and magnetic field generation platforms, effective clinical translation requires holistic consideration of surgical workflows, intraoperative localization, and operator interaction. Integrating these engineering advances into routine clinical practice remains an ongoing challenge. This review provides a comprehensive synthesis of recent advances in MACRs from a clinical perspective, highlighting technologies that accelerate their translation into minimally invasive surgery. The fundamental working principles of MACRs are first outlined, followed by a task- driven categorization of robotic designs. The emerging strategies for intraoperative localization and motion control at varying levels of autonomy are then evaluated. Furthermore, representative applications across physiological systems are summarized. Finally, key challenges are highlighted to elucidate current technological boundaries and provide a roadmap for future research.

## Introduction

Continuum instruments have reshaped minimally invasive surgery (MIS) due to their intrinsic compliance and jointless architectures, allowing navigation through tortuous anatomical pathways, such as complex vasculature and natural luminal environments [1]. However, conventional instruments remain largely passive at their distal ends, relying on proximal push–pull transmission, shaft rotation, and contact forces from anatomical boundaries. Such indirect actuation limits distal dexterity, which may cause procedural complexity and increase the risk of tissue trauma. To overcome these limitations, continuum robots (CRs) have been introduced, integrating embedded onboard actuators such as pneumatic, hydraulic, concentric- tube and tendon- driven mechanisms to achieve active distal steering [2]. Among various designs, magnetically actuated continuum robots (MACRs) have attracted extensive attention. Low- frequency and small- amplitude magnetic fields can traverse biological tissues with minimal attenuation, applying torques and forces directly to the distal tip [3]. This wireless actuation approach displaces the actuation source outside the patient, eliminating the need for complex internal mechanical structures and enabling miniaturization [4–6], enhanced compliance [7–9], and improved steering capabilities [10–12].

MACRs have undergone steady technological maturation over the past 2 decades, as extensively reviewed in Refs. [13–16] and more recently in Refs. [17,18]. Although these pioneering works offer excellent overviews of foundational engineering frameworks, kinematics modeling, and control algorithms, they primarily focus on engineering- centric technical modalities. However, a systematic analysis of how these decoupled engineering breakthroughs map to clinical tasks is still lacking. Promising preclinical demonstrations and early translational efforts have been reported but are far from sufficient. Full clinical deployment requires a holistic consideration of surgical workflows, intraoperative localization, and operator interaction [19]. To accelerate this transition, providing researchers in all disciplines with integrated insights that bridge technological advances and clinical constraints is essential to translate MACRs into practical surgical devices.

The broader category of MACRs can be classified into untethered devices (e.g., wireless soft- bodied robots) and tethered devices (e.g., guidewires, catheters, and endoscopes). Untethered magnetic robots represent a distinct and rapidly evolving research direction with superior access to hard- to- reach anatomical regions [20–23]. However, issues related to functional integration, controllability in dynamic physiological environments, and reliable retrieval remain under investigation [24]. In addition, while tethered magnetic robots encompass both capsule- type devices and slender continuum instruments, tethered capsule robots are beyond the scope of this review. This exclusion is due to their distinct operational modalities, in which the tether often serves as a passive conduit for power, data transmission, or auxiliary working channels, rather than providing structural continuity and mechanical transmission capabilities. This review specifically focuses on slender, high- aspect ratio continuum instruments with inherent structural continuity that leverage established interventional paradigms, which currently offer a more direct path to near- term clinical deployment.

With this defined scope, this review provides a survey of recent MACR advances from a clinical perspective, highlighting the technologies that facilitate their translation into MIS. For a broader background, several representative reviews may be consulted. For example, Abbott et al. [3] thoroughly delineate the mathematical and physical principles governing magnetic actuation, while Nelson et al. [19] provide a detailed account of the historical development and early milestones of the field. The objectives of this review are 3- fold: first, to provide an integrated overview of the latest developments in MACR technology; second, to examine potential clinical applications across human physiological systems while highlighting task- driven design considerations; and third, to analyze existing technical and translational challenges to guide future research. The remainder of this review is organized as follows. Working Principle introduces the fundamental working principles. Robotic Design categorizes robotic designs based on specific clinical tasks. Intraoperative Localization discusses emerging approaches for intraoperative localization, while Control Strategies reviews control strategies according to different levels of autonomy. Potential Applications summarizes potential applications across major human physiological systems. Finally, Conclusion and Outlook highlights open clinical questions and translational considerations.

## Working Principle

This section outlines the fundamental principles of MACR technology to provide a baseline for interdisciplinary readers. The discussion first establishes the mechanisms of magnetic torque and force generation, followed by an analysis of the architectural integration of proximal transmission with distal responsive units. Finally, magnetic field generation platforms (MFGPs) are evaluated with an emphasis on those that have demonstrated clinical or preclinical translational potential.

### Magnetic physics

There are 2 primary interactions when a magnetic element is placed in a magnetic field ( B ), as illustrated in Fig. 1A. Magnetic torque ( $T_{m}$ ): In a uniform magnetic field, the magnetic element experiences a torque that tends to align its magnetic moment ( m ) with the direction of the external magnetic field. Magnetic force ( $F_{m}$ ): In a nonuniform magnetic field, that is, with a magnetic field gradient, the magnetic element is subjected to a translational force that drives it toward regions of higher field intensity. These interactions are governed by the following expressions:

$$\begin{array}{ll} T_{m} & =m\cdot B\\ F_{m} & =\left(m\cdot \nabla \right)B \end{array}$$ (1)

**Fig. 1.**
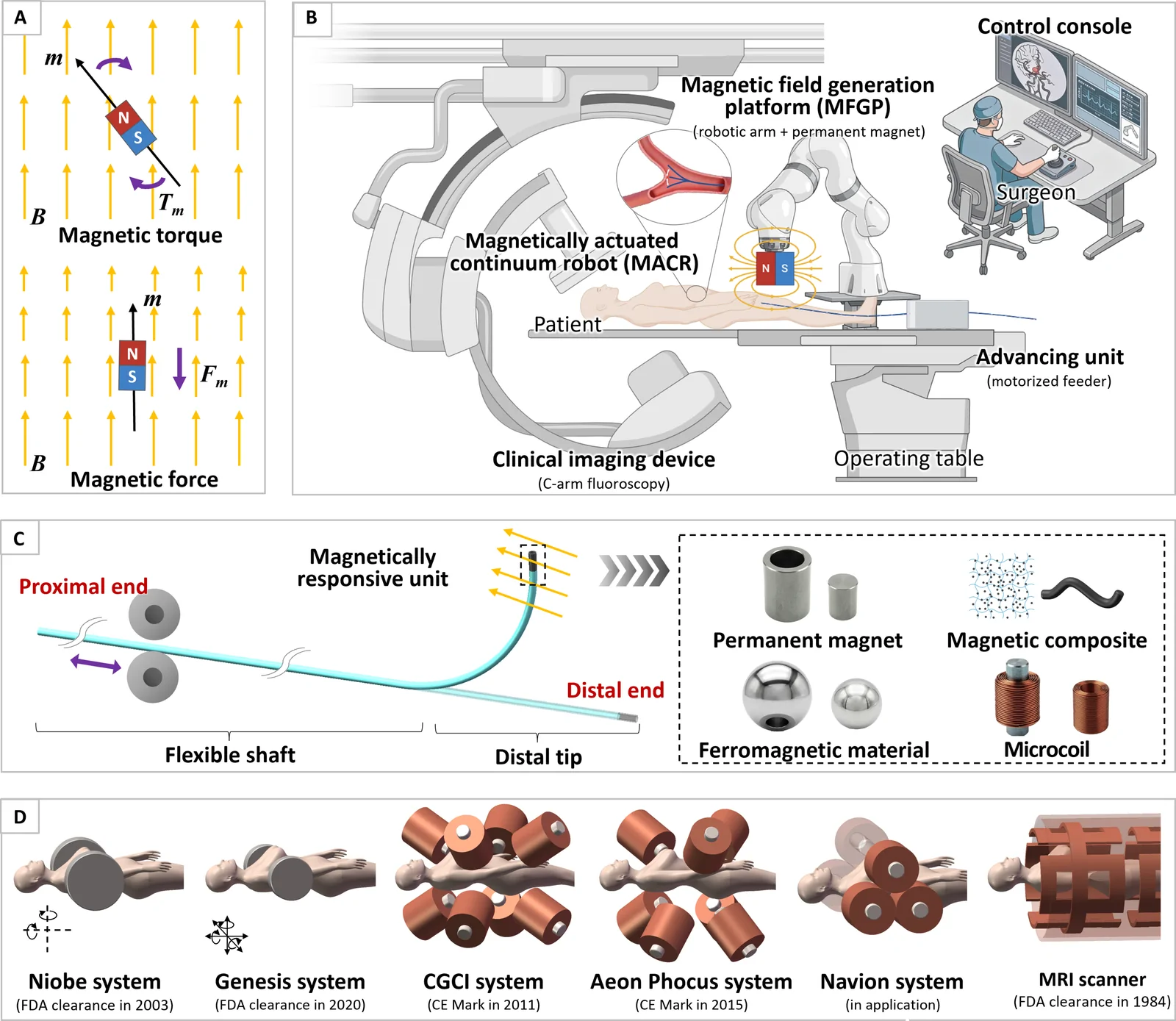
General working principle of magnetically actuated continuum robots (MACRs). (A) Diagram of magnetic interaction, including magnetic torque and force. (B) A typical setup for a clinical scenario, consisting of MACR, magnetic field generation platform (MFGP), clinical imaging device, advancing unit, and control console. (C) The general architecture of the MACR, including flexible body and a magnetically responsive unit. (D) Illustration of magnetic field sources with respect to the patient for clinical and advanced preclinical MFGPs. Panels (A), (C), and (D) are adapted and redrawn from Ref. [15]. Copyright 2023, Wiley.

In clinical scenarios, magnetic torque and force often coexist, and their respective roles are typically prioritized according to the surgical task [25]. For steering through intricate anatomical pathways, magnetic torque is generally the dominant interaction to induce distal tip deformation, while gradient- induced force is minimized or treated as a disturbance [4,26]. In contrast, for traversing large cavities or directly penetrating occlusions, magnetic force often becomes the primary driver [27,28]. These task- specific requirements necessitate magnetic field generation strategies to either decouple or combine these interactions for precise navigation and functional integration [29,30]. For more rigorous and detailed mathematical and physical derivations of magnetic actuation, readers may refer to the foundational work of Abbott et al. [3].

### Fundamentals of MACRs

Most MACRs are operated through a coordinated control loop that includes proximal actuation, external magnetic field steering, and intraoperative feedback. Consequently, a representative clinical or preclinical setup typically comprises a MACR as the core end- effector, an MFGP to produce the required external magnetic field, a clinical imaging device for real- time guidance, an advancing unit for proximal manipulation, and a control console enabling the operator to issue real- time commands. A typical setup is illustrated in Fig. 1B.

Regarding the MACR itself, a typical architecture comprises a flexible body and a distal magnetically responsive unit (Fig. 1C). The flexible body is commonly made from biocompatible polymers (e.g., Pebax, polytetrafluoroethylene, and silicone) and nitinol, providing the necessary flexibility to navigate tortuous pathways while maintaining sufficient structural integrity for push–pull transmission. The distal tip usually has a lower stiffness than that of the flexible body, serving as a deformable substrate for steering. The magnetically responsive unit incorporates magnetic elements that convert external magnetic energy into mechanical work. These magnetic elements are categorized into 4 types based on the material composition:

• Permanent magnets: They are made of rare- earth alloys (e.g., NdFeB, and SmCo) that exhibit high remanence and volumetric torque density [5,10,31,32]. Permanent magnets can be miniaturized while maintaining high performance. However, their inherent rigidity may require consideration of mechanical safety during physical tissue contact. Although previously deemed incompatible with magnetic resonance imaging (MRI) scanners, these materials have been shown to be feasible to use in such environments [33,34].
• Magnetic composites: They are soft matter that comprises magnetic micro/nanoparticles (e.g., NdFeB and iron oxide) embedded within an elastomer matrix (e.g., silicone and hydrogels) [11,12,35,36]. These materials facilitate shape programming and complex soft- bodied deformations. However, their magnetic performance is often constrained by the maximum loading density of the fillers.
• Ferromagnetic materials: They are soft magnetic materials (e.g., Mu- metal, permalloy, and certain stainless steels) that exhibit high magnetic permeability in the external magnetic field but negligible remanence when the field is removed [37,38]. While inherently MRI-compatible, these materials exhibit nonlinear hysteretic behavior and typically generate lower torque density compared to permanent magnets.
• Microcoils: They are small- scale electromagnetic coils connected to an external power source, which enable on- demand adjustment of the magnetic moment via current control [39–41]. The primary advantage lies in their dynamic controllability, although they face challenges related to Joule heating and scaling limitations.

### Magnetic field generation platforms

MFGPs can produce the required magnetic fields for actuation. To meet clinical needs, these platforms should deliver adequate field or gradient intensities within clinically relevant workspaces, while maintaining compatibility with medical imaging devices and surgical workflows. They can be categorized into 2 types based on their field sources:

• Permanent magnet- based MFGPs: These platforms utilize one or more high- grade permanent magnets as the magnetic source. Dynamic field generation is achieved through the mechanical positioning and orientation of the magnets using robotic manipulators or dedicated actuation mechanisms. They can achieve high- field and high-gradient intensities at a relatively low cost without requiring complex power supplies or active cooling, thereby facilitating integration into existing operating rooms. However, several limitations exist, including their always- on magnetic field, intrinsic mechanical inertia during field adjustment, and the potential risk of physical collisions [12,29,30,35,36].
• Electromagnetic coil- based MFGPs: These platforms employ stationary or mobile electromagnetic coils to generate the desired field. For stationary configurations, dynamic adjustment is achieved by precisely regulating the current through each coil, with typical configurations including Helmholtz, Maxwell, and multiaxis coil arrays. These systems exhibit rapid temporal response and high- bandwidth control. Nevertheless, they face engineering challenges such as substantial Joule heating and a large physical footprint. To address these, hybrid and reconfigurable configurations have been explored, including the use of single electromagnetic coils as switchable permanent magnets with on–off capability, and mobile coil arrays that use mechanical mobility to provide expanded workspaces [5,20–31,33,42].

The existing MFGPs have been thoroughly reviewed in Refs. [3,29,30]. Therefore, this review focuses on representative platforms that have reached clinical or advanced preclinical stages, as illustrated in Fig. 1D. Based on these representative systems, Table 1 summarizes the typical platform- level tendencies of permanent magnet- based and electromagnetic coil- based MFGPs. Stereotaxis, Inc. (Missouri, USA) has pioneered the commercialization of MFGPs. The Niobe system (Food and Drug Administration [FDA] clearance in 2003) uses 2 large permanent magnets mounted on rotating bases to steer endocardial ablation catheters [43]. Building upon this, the Genesis system (FDA clearance in 2020) represents an architectural evolution, featuring 2 reduced- size permanent magnets mounted onto customized multiaxis robotic manipulators [44]. This configuration simultaneously minimizes the system footprint and improves maneuverability. By early 2024, these systems had cumulatively supported more than 100,000 clinical procedures worldwide [45]. Beyond clinical platforms, the integration of permanent magnets with commercial robotic manipulators represents another research trend. These systems offer an extendable workspace that covers the entire human body, with preclinical validation performed in in vitro vascular phantoms and in vivo animal models [35,46].

**Table 1.** Comparison of permanent magnet- based and electromagnetic coil- based MFGPs. Note: The entries represent typical platform- level tendencies summarized from representative commercial and advanced preclinical systems [25,35,43,44,46–49]; actual performance depends on system geometry, operating conditions, and source- to- target distance.

Evaluation metric	Permanent magnet- based	Electromagnetic coil- based
Field intensity	Typically high, dependent on magnet size and proximity	Typically moderate and tunable, dependent on coil geometry and heating
Gradient intensity	Typically high, localized near magnet surfaces	Typically moderate and tunable, dependent on coil geometry and heating
Workspace	Typically scalable, kinematically adjustable via a robotic manipulator	Typically fixed, constrained by the static coil geometry
Physical footprint	Typically moderate and compact, concentrated around robotic manipulator	Typically large, requiring bulky coils, power supplies, and cooling systems
Cooling requirement	Generally not required for the magnetic source	Often required under high- current continuous operation
Response speed	Typically lower, limited by mechanical actuation inertia	Typically higher, enabled by electronic current control
Imaging compatibility	Typically provides more open access, dependent on manipulator configuration	May restrict imaging access owing to surrounding coil structures
Manufacturing cost	Typically moderate, governed by magnetic materials and robotic positioning hardware	Typically high, may require additional power electronics, cooling, and control hardware
Clinical deployment	Clinical deployment demonstrated for specific MFGPs, such as Niobe, Genesis	Mostly at the preclinical or early translational stage, such as CGCI, Aeon Phocus, Navion

MFGP, magnetic field generation platform

Meanwhile, electromagnetic coil- based systems are also moving out of the lab and into the clinic. Magnetecs Corp. (California, USA) introduced the Catheter Guidance, Control and Imaging (CGCI) system (CE Mark in 2011), which employs an array of 8 electromagnetic coils in a spherical arrangement around the patient [47]. It provides rapid field generation and flexible control over magnetic fields and gradients by controlling individual coil currents. Later, Aeon Scientific AG (Zurich, Switzerland) developed the Aeon Phocus system (CE Mark in 2015), featuring 7 electromagnetic coils mounted on dual movable frames to target endovascular and endoscopic applications [48]. More recently, MagnebotiX AG (Zurich, Switzerland)translated the Navion system, which uses 3 optimized electromagnetic coils with patented cooling technology, allowing easier deployment in operating rooms while maintaining high field density [25]. It also supports the cooperative deployment of 2 Navion systems when required. Furthermore, standard clinical MRI scanners can be repurposed for magnetic actuation (first FDA clearance in 1984) [49]. They provide high magnetic field and gradient intensities and have integrated real- time imaging capabilities, although the unidirectional static field ( $B_{0}$ ) requires specialized MACR designs to achieve multi-degree- of- freedom (DoF) steering.

## Robotic Design

The design of MACRs has evolved from basic steerability to encompass diverse task- specific functionalities. To systematically review these advances, this section categorizes robotic designs according to their clinical functions. The discussion first summarizes the designs tailored for navigation and access, emphasizing strategies for miniaturization and dexterity enhancement. Next, architectures that enable active adaptation and locking are reviewed, where magnetic moment or stiffness can be modulated intraoperatively. Then, designs specialized for therapeutic intervention and tissue manipulation are outlined to highlight versatile operational capabilities. Finally, the integration of sensing and imaging modalities that provide real-time situational awareness is summarized.

Because the performance metrics reported in the original studies vary substantially across task- specific designs, a fully standardized comparison would produce many unreported entries and could encourage inappropriate direct comparisons. Therefore, Tables 2 to 6 use a category- specific Performance column to report the most relevant and commonly available metrics within each design category. Metrics not reported in the original studies were not inferred.

**Table 2.** Characteristics and applications of representative MACRs for navigation and access employing fundamental design strategies

Ref.	Magnetic component	Actuation system	Distal diameter	Distal length	Performance	Workspace	Application scenario	Validation environment
[31]	PM	EMS	2 mm	25 mm	~20- mm deflection	80 mm × 80 mm × 80 mm	Retrograde intrarenal surgery (RIRS)	In vitro phantom
[33]	PM	EMS	832 μm	80 mm	N/A	Human- scale MRI	Hepatic arteries, carotid artery	In vitro phantom, in vivo animal test
[5]	PM	EMS	500 μm	3.8 mm	132.7° steering	Φ 160- mm sphere	Coronary artery	In vitro phantom
[55]	PM	PMS	2 mm	45 mm	J, C, and S- shapes	N/A	Obstacle avoidance	In vitro phantom
[32]	PM	EMS	~3.2 mm	50 mm	8- to 9- mm deflection	~110 mm × 110 mm	Peripheral artery	In vitro phantom
[10]	PM	EMS	2.6 mm	50 mm	210° steering	200 mm × 200 mm × 400 mm	Cardiac ablation	In vitro phantom, ex vivo organ (breast)
[35]	MC	PMS	400 μm	50 mm	120° steering	Robotic manipulator	Cerebral artery	In vitro phantom, in vivo animal test
[11]	MC	EMS	2 mm	50 mm	180° steering	60 mm × 60 mm × 60 mm	Pancreatic and bile ducts	In vitro phantom
[12]	MC	PMS	1.9 mm	25 mm	~200° steering	700 mm × 500 mm × 500 mm	Vascular navigation	In vitro phantom
[36]	MC	PMS	2 mm	80 mm	Follow- the-leader motion	~100 mm × 100 mm × 100 mm	Bronchial tree	In vitro phantom
[59]	MC	PMS	2 mm	80 mm	C, S, and higher- order shapes, >180°	Robotic manipulator	Obstacle avoidance	N/A
[37]	FM	EMS	2 mm	30 mm	20° steering	Human- scale MRI	Renal artery, iliac artery	In vitro phantom, in vivo animal test
[38]	FM	EMS	2.1 mm	32.7 mm	20.3- mm deflection	Human- scale MRI	Vascular navigation	In vitro phantom
[39]	μC	EMS	2 mm	7- mm coil	~75° steering	Human- scale MRI	Vascular navigation	In vitro phantom
[40]	μC	EMS	4 mm	10.5- mm coil	~45- mm deflection	Human- scale MRI	Cardiac intervention	In vitro phantom
[41]	μC	EMS	1 mm	9.54- mm coil	110° steering	Animal- scale MRI	Vascular navigation	In vitro phantom

MACRs, magnetically actuated continuum robots; PM, permanent magnet; MC, magnetic composite; FM, ferromagnetic material; μC, microcoil; PMS, permanent magnet- based system; EMS, electromagnetic- based system; MRI, magnetic resonance imaging.

### MACRs for navigation and access

This section first reviews the fundamental MACR design strategies, categorized by the type of magnetic element, as summarized in Table 2. The most prevalent approach involves integrating a permanent magnet at the distal tip [5,10,31–33,50–55]. For example, Lin et al. [31] developed a millimetric robot comprising a polydimethylsiloxane (PDMS) elastic rod and 3 embedded stacked magnets, which support high- precision steering and multidirectional navigation in constrained environments (Fig. 2A). Azizi et al. [33] proposed a submillimetric robot consisting of a guidewire and an attached magnet. Actuated by directional pulling forces generated by the fringe field of a clinical MRI scanner, this robot achieves remote navigation in the complex distal vasculature. In addition to single-magnet designs, robots with multiple magnets have been investigated. Jeon et al. [5] introduced a submillimetric robot featuring a soft- bodied tip embedded with 2 discrete magnets, allowing for large- angle deformation (Fig. 2B). Beyond standard parallel magnetization, Lin et al. [55] developed a millimetric robot employing magnets with antiparallel magnetization, achieving multimode deformations that include the C- shape, the S- shape, and the J- shape. This robot further improves dexterity and obstacle avoidance. Besides conventional shaft configurations such as guidewires and catheters, alternative deformable architectures have been explored, such as laser- cut flexural patterns and interlocking ball- joint mechanisms [10,32]. These designs demonstrate high kink resistance and structural stability while maintaining low bending stiffness, enabling flexible parametric and customizable designs.

**Fig. 2.**
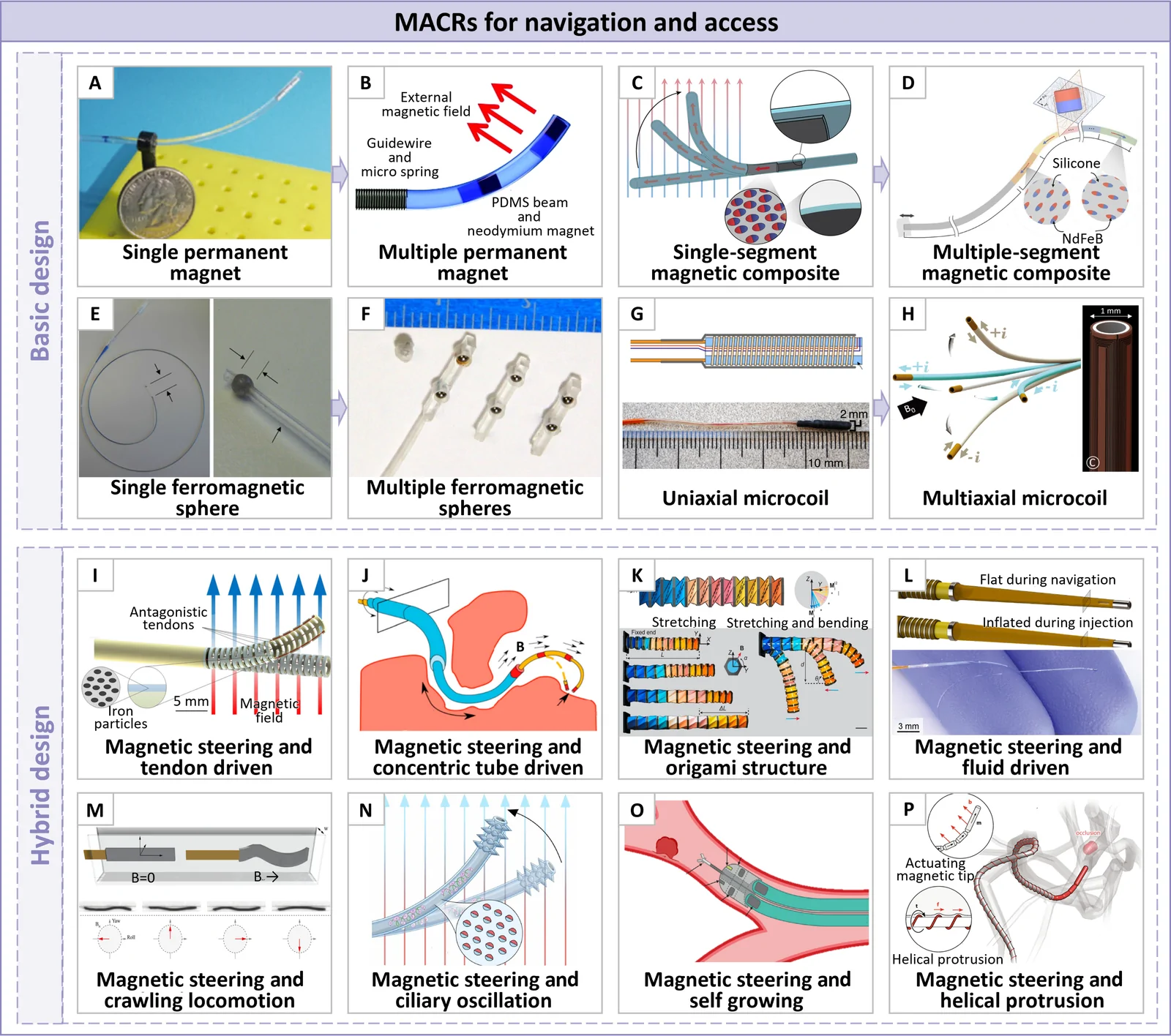
Representative magnetically actuated continuum robots (MACRs) for navigation and access. (A to H) MACRs employing fundamental design strategies: (A) A single permanent magnet. Reproduced from Ref. [31]. Copyright 2021, Wiley. (B) Two permanent magnets. Reproduced from Ref. [5]. Copyright 2019, Mary Ann Liebert. (C) A magnetic composite. Reproduced from Ref. [4]. Copyright 2019, AAAS. (D) Modular axial magnetization. Reproduced from Ref. [59]. Copyright 2025, IEEE. (E) A single ferromagnetic sphere. Reproduced from Ref. [37]. Copyright 2010, ESMRMB. (F) Two ferromagnetic spheres. Reproduced from Ref. [38]. Copyright 2011, American Association of Physicists in Medicine. (G) A uniaxial microcoil. Reproduced from Ref. [39]. Copyright 2014, RSNA. (H) A biaxial microcoil. Reproduced from Ref. [41]. Copyright 2022, Wiley. (I to P) MACRs employing hybrid design strategies: (I) Integration of magnetic steering and tendon- driven actuation. Reproduced from Ref. [61]. Copyright 2021, Wiley. (J) Integration of magnetic steering and concentric- tube actuation. Reproduced from Ref. [62]. Copyright 2022, SAGE. (K) Integration of magnetic steering and an origami structure. Reproduced from Ref. [63]. Copyright 2021, NAS. (L) Integration of magnetic steering and fluid- driven actuation. Reproduced from Ref. [64]. Copyright 2025, AAAS. (M) Integration of magnetic steering and crawling locomotion. Reproduced from Ref. [65]. Copyright 2022, Wiley. (N) Integration of magnetic steering and artificial- cilia oscillation. Reproduced from Ref. [8]. Copyright 2025, AAAS. (O) Integration of magnetic steering and self- growing capability. Reproduced from Ref. [66]. Copyright 2026, Wiley. (P) Integration of magnetic steering and helical protrusion structure. Reproduced from Ref. [9]. Copyright 2024, AAAS.

Magnetic composite- based MACRs have seen rapid advancement in recent years [4,11,12,35,36,56–59]. For example, Kim et al. [4,35] presented a submillimetric robot using a mixture of thermoplastic polyurethane or PDMS and NdFeB microparticles with a self- lubricating hydrogel layer coating. The overall structure is axially magnetized, demonstrating successful navigation through tortuous neurovascular pathways (Fig. 2C). Wang et al. [56] later developed a computational method that can automatically refine the magnetization and material distributions, further optimizing the performance. In addition to uniform axial magnetization, multidirectional magnetization strategies have been devised to enhance steerability. For continuously changing magnetization, Lloyd et al. and Zhang et al. [11,12] proposed millimetric robots in which the continuum structure is programmed with predefined curved shapes prior to magnetization, effectively extending the achievable bending angle. For discretely changing magnetization, Pittiglio et al. [36] developed a millimetric robot consisting of alternating magnetic composite segments and soft elastomer segments. By applying patient- specific magnetization to each segment, the robot achieves follow- the- leader navigation tailored to complex anatomy. Cao et al. [59] proposed a millimetric robot composed of modularized magnetic composite segments, providing theoretical guidance for improving spatial deformation precision (Fig. 2D).

The subsequent categories focus on designs primarily tailored for MRI- integrated environments, starting with ferromagnetic materials [37,38]. Zhang et al. [37] developed a millimetric robot by integrating a ferromagnetic sphere into a flexible silicone tube, enabling directional deflection within standard MRI gradient fields (Fig. 2E). Later, Gosselin et al. [38] introduced a multisphere configuration that nearly doubles the deflection amplitude without increasing the device diameter (Fig. 2F). Microcoil- based actuation offers an alternative for MRI integration [39–41,60]. Losey et al. [39] demonstrated a millimetric robot with a tip- mounted microcoil, which interacts with the static magnetic resonance field and performs planar steering (Fig. 2G). Ali et al. [40,60] expanded the microcoil to a triaxial configuration, allowing spatial steering control. Traditional coils are typically manually wound and hard to miniaturize. Phelan et al. [41] introduced a microwinding technique to fabricate quad- configuration coils on polyimide mandrels, achieving 3D bending within a minimized package (Fig. 2H).

To further improve accessibility and dexterity, hybrid designs have been explored, which are summarized in Table 3. Zhang et al. [61] developed a millimetric robot by coating a magnetic composite layer onto a 3D- printed, notched skeleton configured for tendon actuation, achieving large- angle steering and micron- scale positioning accuracy (Fig. 2I). Peyron et al. [62] presented a millimetric robot that combined concentric tube mechanics and a distal permanent magnet, achieving enhanced tip dexterity and active stability control (Fig. 2J). Wu et al. [63] developed a millimetric robot based on a magnetically controlled Kresling origami structure, achieving multimodal deformations that include stretching, and omnidirectional bending and twisting (Fig. 2K).

**Table 3.** Characteristics and applications of representative MACRs for navigation and access employing hybrid design strategies

Ref.	Driving component	Distal diameter	Distal length	Performance	Application scenario	Validation environment
[61]	MC layer coating + tendon- driven skeleton	3 mm	20 mm	100° steering, 2- μm positioning	Drug injection, diseased tissue ablation, nasopharyngeal swab	In vitro phantom
[62]	PM tip + concentric tubes	4- mm PM, 1.07- mm tube	116 mm	160° steering, 5.4- mm radius of curvature	Olfactory cells inspection, middle- ear surgery	N/A
[63]	PM tip + Kresling units	~10 mm	~50 mm	Stretching, folding, bending, twisting motion	Endoscopy, intubation, catheterization	N/A
[64]	PM tip + flow- driven inflatable tube	180 μm	80 mm	Tracking 0.69- mm radius of curvature	Superselective arterial embolization	In vitro phantom, in vivo animal test
[65]	MC tip + polymer tether	400 μm × 200 μm	3.5 mm	Shift between flow- assisted and magnetic crawling locomotion	Submillimeter arteries with impaired flow	In vitro phantom, ex vivo organ (heart)
[8]	MC tip + integrated artificial- cilia array	400- μm central, 270- μm cilia	15 mm	90° steering, reducing friction by two- thirds	Traversal of narrow and tortuous pathways	In vitro phantom, ex vivo organ (bile duct), in vivo animal test
[66]	PM tip + pneumatic thin- film eversion	6.3 mm PM, 7- mm tube	~90 mm	>90° steering, 34- mm bending radius, minimize tissue stress	Endoluminal navigation	In vitro phantom
[9]	PM tip + backbone with helical protrusion	800 μm	14 mm	>90° steering, translates rotation to forward motion at every point of contact	Cerebral/brain arteries	In vitro phantom, in vivo animal test

MACRs, magnetically actuated continuum robots; PM, permanent magnet; MC, magnetic composite; N/A, not applicable

Beyond the hybrid steering strategy, innovative propulsion mechanisms have been developed to overcome the limitations of traditional proximal push–pull transmission [7–9,64–66]. Pancaldi et al. [7,64] proposed a submillimetric robot that harvests hydrodynamic energy from blood flow in conjunction with magnetic steering, allowing navigation and embolization in deep and tortuous arterial branches (Fig. 2L). They also presented a submillimetric robot that integrates flow sensors with a magnetically actuated soft- bodied tip. This robot is capable of autonomously switching to an active crawling propulsion mode under a rotating magnetic field when operating in impaired flow conditions (Fig. 2M) [65]. Yang et al. [8] developed a millimetric foxtail grass- inspired robot with magnetically actuated artificial cilia and a slippery coating, markedly reducing navigation friction through magnetic oscillation (Fig. 2N). Yanez Trujillo et al. [66] introduced a millimetric vine- inspired robot using tip eversion, enabling low- stress navigation through self- growing motion that avoids conventional entirety sliding (Fig. 2O). Dreyfus et al. [9] developed a submillimetric robot integrated with a helical protrusion structure, which converts proximal rotational motion into forward propulsion, overcoming the pushability limitations of highly flexible devices in tortuous vasculature (Fig. 2P).

### MACRs for adaptation and locking

To enhance morphological adaptability, MACRs with adjustable magnetization have been developed [67,68]. These mechanisms enable intraoperative shape programming, allowing the robot to reconfigure its bending profiles to accommodate complex anatomical environments. Cao et al. [67] proposed a millimetric robot with 2 programmable proximal actuating units that uses integrated heating coils to trigger the phase transition of a low- melting- point polymer (LMPP). This allows the magnetization direction of the programmable proximal actuating unit to be reconfigured under external magnetic fields in the molten state and subsequently locked upon solidification (Fig. 3A). Qin et al. [68] introduced a millimetric robot that features a dual- coil structure at the tip, enabling precise current- mediated magnetization and near- instantaneous deenergization (Fig. 3B).

**Fig. 3.**
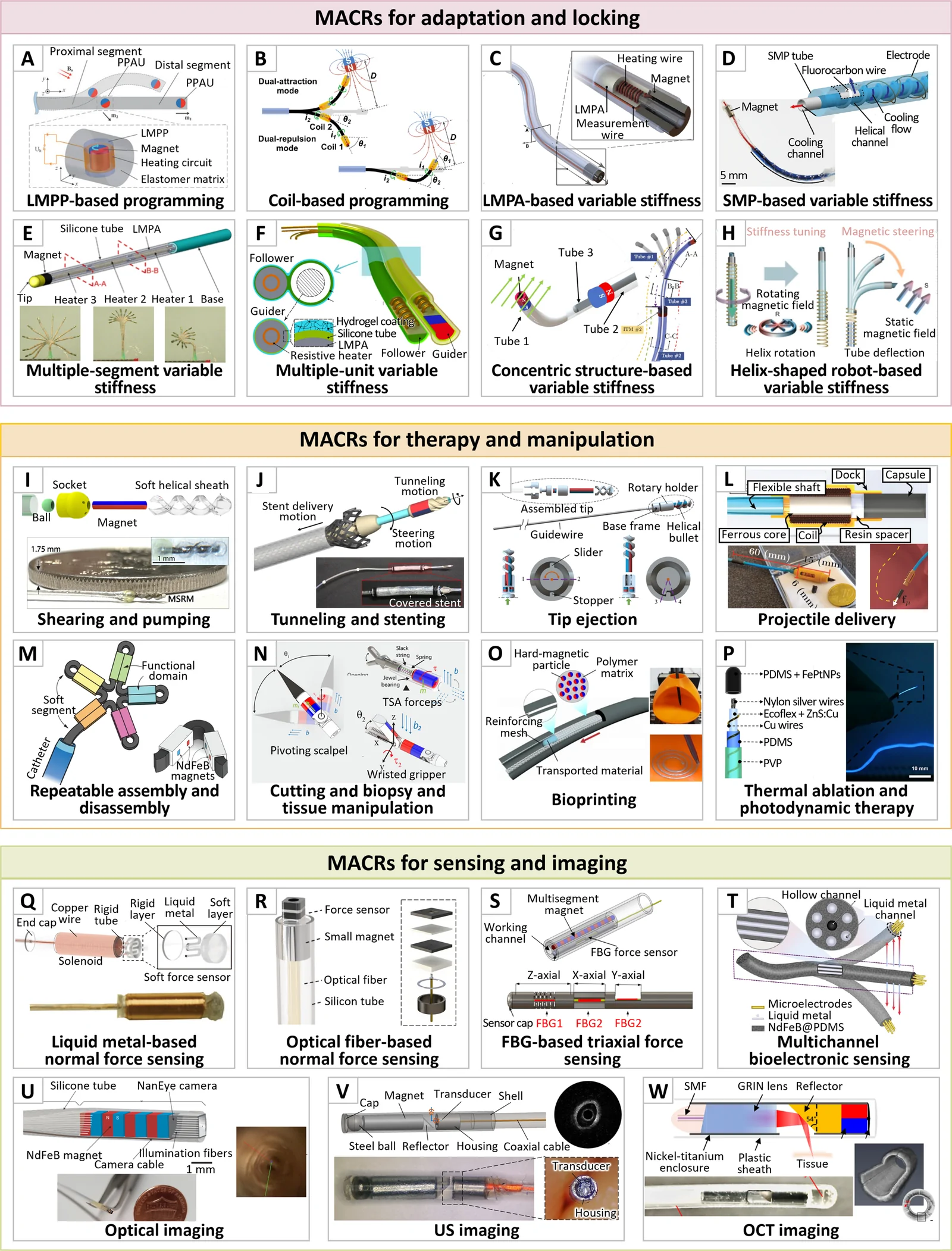
Representative magnetically actuated continuum robots (MACRs) with task- specific functionalities. (A to H) MACRs for adaptation and locking: (A) Magnetic catheter with intraoperative magnetic moment programming. Reproduced from Ref. [67]. Copyright 2023, Mary Ann Liebert. (B) Magnetic guidewire with dual coils for adaptive curvature steering. Reproduced from Ref. [68]. Copyright 2026, Wiley. (C) Magnetic catheter with variable stiffness using low- melting- point alloy (LMPA). Reproduced from Ref. [69]. Copyright 2021, Wiley. (D) Magnetic catheter with variable stiffness using shape memory polymer (SMP). Reproduced from Ref. [72]. Copyright 2024, Wiley. (E) Magnetic catheter with multiple variable stiffness segments. Reproduced from Ref. [48]. Copyright 2020, Wiley. (F) Magnetic catheter with multiple variable stiffness units. Reproduced from Ref. [74]. Copyright 2024, Nature. (G) Magnetic catheter with variable stiffness based on concentric structure. Reproduced from Ref. [77]. Copyright 2024, IEEE. (H) Magnetic catheter with variable stiffness based on helix- shaped robot. Reproduced from Ref. [80]. Copyright 2026, Nature. (I to P) MACRs for therapy and manipulation: (I) Magnetic catheter with soft rotatable tip. Reproduced from Ref. [83]. Copyright 2025, AAAS. (J) Magnetic catheter with steering, tunneling, and stent delivery. Reproduced from Ref. [84]. Copyright 2020, IEEE. (K) Wired magnetic microrobot with ejectable tip. Reproduced from Ref. [85]. Copyright 2023, IEEE. (L) Magnetic catheter for projectile delivery. Reproduced from Ref. [27]. Copyright 2021, IEEE. (M) Magnetic robot with self- folding chains with reconfigurable shapes and functionalities. Reproduced from Ref. [88]. Copyright 2023, Nature. (N) Magnetic dexterous tools for minimally invasive operation. Reproduced from Ref. [90]. Copyright 2025, AAAS. (O) Magnetic catheter for minimally invasive bioprinting. Reproduced from Ref. [91]. Copyright 2021, Nature. (P) Optoacoustically augmented magnetic guidewire. Reproduced from Ref. [6]. Copyright 2026, AAAS. (Q to W) MACRs for sensing and imaging: (Q) Magnetic catheter with normal force sensing using liquid metal. Reproduced from Ref. [92]. Copyright 2024, IEEE. (R) Magnetic catheter with normal force sensing using optical fiber. Reproduced from Ref. [93]. Copyright 2025, Elsevier. (S) Magnetic catheter with triaxial force sensing using fiber Bragg grating (FBG). Reproduced from Ref. [94]. Copyright 2025, IEEE. (T) Magnetic catheter for multichannel bioelectronic sensing. Reproduced from Ref. [95]. Copyright 2025, Nature. (U) Magnetic bronchoscope. Reproduced from Ref. [96]. Copyright 2018, World Scientific. (V) Dual- mode magnetic intravascular ultrasound catheter robot. Reproduced from Ref. [26]. Copyright 2024, IEEE. (W) Motor- free magnetic optical coherence tomography (OCT) catheter robot. Reproduced from Ref. [97]. Copyright 2024, Nature.

To address the inherent trade- off between dexterous obstacle avoidance and precise manipulation, variable- stiffness MACRs have been proposed. One mainstream approach is to utilize phase- change materials [48,69–75]. For example, Lussi et al. [69] developed a submillimetric robot integrated with a heating wire and encapsulated low- melting- point alloy, producing a 400- fold variation in structural stiffness by changing radial thermal gradients (Fig. 3C). Mattmann et al. [70] presented a millimetric robot integrated with a heating wire and shape memory polymer, achieving a 93- fold stiffness variation. The group subsequently integrated helical cooling channels into the MACR, accelerating the stiffness transition speed by 66- fold (Fig. 3D) [72]. Piskarev et al. designed a millimetric robot using conductive shape memory polymer that does not require additional heating wire, achieving a 21- fold stiffness variation. The robot also integrates temperature sensing capabilities, facilitating the implementation of closed- loop stiffness control [73]. Based on independently controlled electrothermal actuation, multisegment designs have been proposed to enable localized compliance control and morphological locking, thus facilitating complex S- shaped navigation (Fig. 3E) [48]. Furthermore, multiunit designs have been developed, where one unit serves as the guider and another as the follower. The robot utilizes periodic phase- transition cycles to enable autonomous shape formation independent of environmental interaction (Fig. 3F) [74,75].

Another alternative is structural mechanical variation, which offers near- instantaneous stiffness adjustment in response to control inputs [76–80]. For example, Li et al. [76] developed a millimetric robot with 4 discrete stiffness modes. The system enables dynamic switching through telescopic motion of concentric catheter and guidewire. Li et al. [77,78] presented a millimetric robot with continuously variable stiffness, whose adjustment is achieved by adjusting the overlap of 3 concentric components (Fig. 3G). Sun et al. [79] developed a millimetric robot utilizing a vacuum- driven fiber jamming mechanism. By incorporating up to 1,000 ultrathin fibers (50- μm diameter) within a silicone sleeve, the system achieves a 6.5- fold stiffness change. Liu et al. [80] introduced a submillimetric architecture that can be integrated into commercial microcatheters, achieving up to a 40- fold stiffness tuning and precise steering via a helix- shaped magnetic soft robot (Fig. 3H). The representative MACRs for adaptation and locking are summarized in Table 4.

**Table 4.** Characteristics and applications of representative MACRs for adaptation and locking

Ref.	Magnetic component	Distal diameter	Distal length	Adjustment mechanism	Performance	Application scenario	Validation environment
[67]	PM	2 mm	40 mm per segment	Thermal phase- change LMPP + heating circuit, reorienting internal magnets	J, C, and S shapes, ~120- s magnetization tuning, 125.6° steering	Obstacle avoidance	In vitro phantom
[68]	μC	4.6 mm	30- mm spacing	Real- time current- adjusted polarity	Dual- attraction, dual-repulsion, spoon-shaped modes, 90° steering, 5- mm bending radius	Vascular navigation	In vitro phantom
[69]	PM	900 μm	30 mm	Thermal phase- change LMPA + heating circuit	400- fold stiffness tuning, 10- s heating, 30- s cooling	Ophthalmic surgery	In vitro phantom
[70]	PM	1.3 mm	35 mm per segment	Thermal phase- change SMP + heating circuit	93- fold stiffness tuning, 9- s heating, 60- s cooling	Transluminal procedures, endovascular procedures	In vitro phantom
[72]	PM	2.3 mm	25 mm per segment	Thermal phase- change SMP + heating circuit + water cooling	66- fold stiffness tuning, 13- s heating, 4.4- s cooling, 127° steering	Cardiac ablation	In vitro phantom
[73]	PM	2.0 mm	44 mm per segment	Direct electrothermal Joule heating CSMP	21- fold stiffness tuning, 27- s heating, 107- s cooling	Cardiac ablation	In vitro phantom
[48]	PM	2.33 mm	15 mm per segment	Thermal phase- change LMPA + heating circuit	Selective softening of serial segments, 15- s heating, 80- s cooling	Cardiac ablation, gastrointestinal endoscopy	N/A
[74]	PM	~3–4 mm	40 mm	Thermal phase- change LMPA + heating circuit	Apical extension via cyclic advancing mechanism, 5- s heating, 15- s cooling	Transluminal procedures, endovascular procedures	In vitro phantom, ex vivo organ (stomach), in vivo animal test
[76]	PM	3.0 mm	N/A	Telescopic motion of concentric catheter and guidewire	Four working modes, 180° steering	Glioma treatment	In vitro phantom
[77]	PM	3.5 mm	60–100 mm	Telescopic motion of 3 concentric tubes	21- fold stiffness tuning ratio, >120° steering	Percutaneous coronary intervention (PCI)	In vitro phantom
[79]	PM	2.3 mm	40 mm per segment	Vacuum- driven fiber jamming	6.5- fold stiffness tuning, 300- ms stiffness change, 107° steering	Cardiac ablation	In vitro phantom
[80]	MC	500 μm–2 mm	3.8–6 mm	Adjust the overlap of rigid helix and soft microtube	40- fold stiffness tuning, 10- fold stiffness change in 300 ms, >120° steering	Transluminal procedures, endovascular procedures	In vitro phantom, ex vivo organ (oviduct), in vivo animal test

MACRs, magnetically actuated continuum robots; PM, permanent magnet; MC, magnetic composite; μC, microcoil; LMPA, low- melting- point alloy; SMP, shape memory polymer; CSMP, conductive shape memory polymer. Heating and cooling times are measured in air.

### MACRs for therapy and manipulation

One of the most representative clinical applications for MACRs is intravascular recanalization, such as thrombectomy and atherectomy, which draws inspiration from established commercial devices (e.g., Jetstream atherectomy device, Phoenix atherectomy system, and Rotablator system) [28,54,81–84]. For example, Nguyen et al. [81] developed a millimetric robot for percutaneous revascularization in chronic total occlusions, featuring a rigid magnetic helical effector integrated at the distal tip via a microspherical joint. The external rotating magnetic field drives the helical structure in a corkscrew motion, generating sufficient drilling torque and force to disrupt and fragment occlusive thrombi. Zhang et al. [83] proposed a submillimetric robot featuring a soft magnetic helical effector and a miniaturized ball joint. This robot not only accelerates thrombolytic diffusion through vortex- induced transport but also mechanically breaks down clots via physical shear stress, while enabling the retrieval of clot debris (Fig. 3I). Lee et al. [84] reported a multifunctional millimetric robot equipped with a magnetic drill tip and a magnetic joint, capable of remote active steering, high- speed rotational tunneling, and stent delivery under controlled magnetic fields (Fig. 3J).

Another emerging category involves modular and separable architectures, which address the contradiction between the navigation constraints of tethered robots and the retrieval complexities of untethered robots [27,46,85–87]. For example, Yang et al. [85] proposed a millimetric robot with active steering and an ejectable tip. The robot utilizes an array of 3 permanent magnets, where the coupling states are controlled by internal magnetic interactions and external field triggers (Fig. 3K). Sa et al. [79] developed a millimetric robot with similar functionality, employing a screw- coupled mechanism for connection and separation. Sikorski et al. [27] investigated a millimetric robot integrated with a distal microcoil, allowing high- precision steering and serving as a deployment platform for controlled ejection and retrieval of the loaded magnetic capsule (Fig. 3L).

In addition, MACRs integrated with versatile manipulation capabilities have been reported [88–90]. Gu et al. [88] developed a millimetric robot comprising self- assembling magnetic soft chains (MaSoChains), which facilitates reversible in situ assembly through simple proximal push–pull motions. This allows the formation of functional structures, such as a catheter with 360° reachable coverage and a gripper for transport and release (Fig. 3M). Fan et al. [89] introduced a submillimetric robot integrating a cat- tongue- inspired microbarb array, which achieves decoupled control of navigation and micromanipulation, such as clot removal and liquid biopsy. He et al. [90] proposed a class of millimetric microsurgical tools for neurosurgical applications, such as the treatment of deep- brain tumors and epilepsy. These include a 2- DoF wrist gripper, a 1- DoF pivoting scalpel, and a TSA forceps, allowing precise cutting, tissue manipulation, and biopsy (Fig. 3N).

Finally, MACRs are increasingly integrating specialized physiological functions [6,91]. Zhou et al. [91] presented a millimetric robot for minimally invasive in situ bioprinting, composed of NdFeB- PDMS composite and polylactide fiber- reinforcing mesh, ensuring high- precision magnetic control and channel stability during high- pressure extrusion (Fig. 3O). Wang et al. [6] developed a submillimetric robot that integrates nonionizing optoacoustic (OA) imaging with magnetic steering. By incorporating FePt nanoparticles and electroluminescent fibers, this device enables in situ radiofrequency- induced thermal ablation and photodynamic therapy (Fig. 3P). The representative MACRs for therapy and manipulation are summarized in Table 5.

**Table 5.** Characteristics and applications of representative MACRs for therapy and manipulation

Ref.	Magnetic component	Distal diameter	Distal length	Working mechanism	Performance	Application scenario	Validation environment
[81]	PM	1.6 mm	9.5 mm	Helical tip performs drilling and steering	0.8- N force and 0.1125-mN·m torque at 100 Hz	Occlusive vascular diseases	In vitro phantom, in vivo animal test
[83]	PM	800 μm	3 mm	Soft tip acts as micropump to deliver drug and reduce friction	Hybrid treatment clears blood clots 300% faster than tPA drug alone	Vascular navigation for prompt access and treatment	In vitro phantom, ex vivo organ (placenta), in vivo animal test
[84]	PM	3.5 mm	N/A	Magnetic joint and tension spring mechanism	Perform steering, tunneling and stent deployment, full deployment takes ~0.1 s	Occlusive vascular diseases	In vitro phantom
[85]	PM	2.8 mm	12.5 mm	Rotating locker and magnetic ejector	Perform steering, instantaneous separation, and wireless propulsion	Vascular navigation for tortuous and deep lesions	In vitro phantom
[86]	PM	~2.33 mm	~10 mm	Separable and recombinable screw- coupled mechanism	0.186- mN·m magnetic torque, 90.1- mN suction force, separation in 20 s	Occlusive vascular diseases	In vitro phantom, in vivo animal test
[27]	PM + μC	6 mm	15 mm	Projectile delivery and retrieval via current- adjusted polarity	0.37- mm catheter tracking accuracy, 0.82- mm capsule targeting accuracy	Access to deep- seated locations	In vitro phantom
[88]	PM	3 mm × 3 mm	N/A	Self- folding MaSoChains array	Reconfigurable shapes and functionalities	Surgery in open cavities	In vitro phantom
[89]	MC	500 μm	20 mm	Orthogonal magnetization of fiber body and distal thin- film microgripper	Independent navigation and cat- tongue-inspired barb claw manipulation	Endovascular procedures	In vitro phantom
[90]	PM	3.2 mm	15–20 mm	Wristed mechanisms and magnetic twisted string actuator	45° angle gripper jaw, 60° angle wrist/scalpel, 0.7° scalpel accuracy	Endoscopic neurosurgery	In vitro phantom, in vivo animal test
[91]	MC	4–7 mm	80–110 mm	Fiber- reinforced soft catheter	0.53- mm printing resolution, 4% channel lateral expansion, 98.6% cell survival rate	In situ bioprinting	In vitro phantom, in vivo animal test
[6]	MC	~250 μm	up to 10 m	Incorporates electroluminescent and photothermal materials	690- mW luminescence power, tip heated to 82° within 10 s	Hyperthermia tumor ablation, photodynamic therapy (PDT)	In vitro phantom, ex vivo organ (heart)

MACRs, magnetically actuated continuum robots; PM, permanent magnet; MC, magnetic composite; μC, microcoil; tPA, tissue plasminogen activator

### MACRs for sensing and imaging

To improve procedural safety and facilitate closed- loop control, recent research has focused on integrating miniaturized force sensors to provide real- time haptic feedback [92–94]. Wang et al. [92] developed a millimetric robot featuring a solenoidal tip and a liquid metal- based soft force sensor. By measuring resistance variations induced by deformation, this sensor provides real- time normal force feedback with a resolution of 0.1 N (Fig. 3Q). Li et al. [93] proposed a more compact millimetric magnetic catheter that incorporates a permanent ring- shaped magnet and an optical fiber- based Fabry–Perot cavity sensor. This design offers high- precision contact force feedback with a sensitivity of 0.69 nm/kPa, utilizing the deformation- induced shift of interference peaks detected by a spectrometer (Fig. 3R) [93]. Fu et al. [94] introduced a millimetric robot that integrates a permanent magnet with a fiber Bragg grating- based triaxial force sensor. This robot delivers triaxial haptic feedback, achieving an axial resolution of 3.68 mN and lateral resolutions of less than 1 mN. Three fiber Bragg gratings with different center wavelengths are inscribed onto a single optical fiber, which is further embedded within a custom- designed flexure- beam frame. This configuration enables the decoupled measurement of triaxial contact forces by monitoring wavelength shifts induced by the mechanical strain (Fig. 3S).

Beyond physical force sensing, advances in 4D printing have enabled multiplexed biochemical monitoring. Yang et al. introduced a millimetric robot fabricated using a specialized 1- step 4D multichannel printing technique. By embedding 6 liquid metal microchannels within a flexible ferromagnetic polymer matrix, the robot maintains stable electrical conductivity and high flexibility under extreme deformations. This robot demonstrates active steering and simultaneous in situ sensing of 8 biochemical analytes, including metabolites, ions, and pH levels (Fig. 3T) [95].

To address clinical requirements for in situ, real- time, and multiscale diagnostics in MIS, researchers have integrated various imaging modalities into MACRs [26,96,97]. Edelmann et al. developed a millimetric endoscopic robot equipped with 4 permanent magnets, a miniature complementary metal- oxide-semiconductor camera, and a series of 50 optical fibers, enabling concurrent magnetic steering and high- performance optical imaging (Fig. 3U) [96]. Yang et al. [26] presented a millimetric intravascular ultrasound (US) robot consisting of an acoustic-magnetic tip with a radially magnetized cylindrical magnet, a 30- MHz single- element transducer, and a 45° reflector. This robot enables active steering in navigation mode and shaft- free acoustic beam scanning in imaging mode, providing B- mode US images with deep- tissue information for diagnosing atherosclerotic plaques (Fig. 3V). Yuan et al. [97] proposed a millimetric optical coherence tomography robot featuring a gradient- index lens- based optical assembly. Using a superluminescent diode with a central wavelength of 842 nm as a broadband light source, this robot provides ultrahigh-resolution images to identify mucosal epithelial abnormalities that could support early- stage cancer screening (Fig. 3W). The representative MACRs for sensing and imaging are summarized in Table 6.

**Table 6.** Characteristics and applications of representative MACRs for sensing and imaging

Ref.	Magnetic component	Distal diameter	Distal length	Working mechanism	Performance	Application scenario	Validation environment
[92]	μC	7.2 mm	23- mm coil, 3.4- mm sensor	Pressure- induced resistance changes in a liquid- metal- filled microchannel	35° steering, 0.1- to 0.6- N force sensing range, 1- Hz response, 0.1- N sensitivity (*Z*)	Cardiac ablation	In vitro phantom
[93]	PM	2.5 mm	2.5- mm magnet, 2.1- mm sensor	Cavity length of integrated optical fiber Fabry–Perot sensor varies under pressure	126° steering, 0- to 1- N force sensing range, 0.69 nm·kPa^-1^ sensitivity (*Z*)	Transbronchial microwave ablation, cardiac ablation	In vitro phantom, ex vivo tissue (heart)
[94]	PM	5 mm	46- mm catheter, 18- mm sensor	Bragg wavelengths of flexible structure- embedded FBGs shift under pressure	90° steering, 0.71- mN (*X*), 0.52- mN (*Y*), and 3.68- mN (*Z*) resolution	Tactile palpation- based tumor/nodule diagnosis	In vitro phantom, ex vivo tissue (kidney)
[95]	MC	2.5 mm	up to ~1.25 m	4D- printed multichannel sheath, embedded with liquid metal and connected to microelectrodes	63.6° steering, multianalyte sensing (Crea, UA, $H_{2}O_{2}$ , Ep, $K^{+}$ , ${\mathrm{Na}}^{+}$ , ${\mathrm{Ca}}^{2+}$ , pH)	In situ multiplexed biosensing	In vitro phantom, ex vivo organ (renal vein and intestine), in vivo animal test
[96]	PM	2 mm	5.5- mm rigid segment, 300- mm flexible segment	Model- free visual servoing using an estimation- based control loop	250 × 250 pixels camera resolution, 50 optical fibers for illumination	Bronchoscopic inspection	In vitro phantom
[26]	PM	1.3 mm	~8.5 mm rigid segment, 30-mm flexible segment	Magnetic rotor and reflector for motor- free acoustic beam scanning	~75° steering, 30 MHz acoustic frequency, 26- mm imaging depth	Intraluminal US imaging	In vitro phantom, ex vivo organ (artery)
[97]	PM	1.3 mm	7- mm rigid segment, 30- to 60- mm flexible segment	Magnetic rotor and reflector for motor- free OCT scanning	110° steering, 800- nm SD- OCT engine, 2.4 and 7.6-μm axial and lateral resolution	Intraluminal microscopic mucosal examination	In vitro phantom, ex vivo organ (trachea, pancreatic duct), in vivo animal test

MACRs, magnetically actuated continuum robots; PM, permanent magnet; MC, magnetic composite; μC, microcoil; FBG, fiber Bragg grating; OCT, optical coherence tomography; SD- OCT, spectral- domain optical coherence tomography

## Intraoperative Localization

High- precision intraoperative localization remains a bottleneck that hinders the clinical translation of MACRs. Real- time pose acquisition is indispensable to achieve robust closed- loop control, ensure procedural safety, and mitigate the risk of iatrogenic tissue trauma. This section categorizes state- of- the- art in vivo localization technologies into 2 primary categories: medical imaging- based approaches, which leverage conventional and emerging clinical imaging equipment for global feedback; and sensor- based methods, which utilize integrated miniaturized sensing elements to provide pose feedback. The comparison of these intraoperative localization modalities is summarized in Table 7.

**Table 7.** Comparison of intraoperative localization modalities for MACRs, including medical imaging- based and sensor- based methods. Note: Spatial and temporal specifications compiled in this table are based on standard clinical workflows and state- of- the- art research.

Modality	Accuracy	Update Rate	Penetration	Radiation	Magnetic actuation compatibility	Closed- loop suitability
X- ray fluoroscopy	High, ~100–200 μm	High, ~7.5–30 Hz	Deep, full- body penetration	Ionizing radiation	High, immune to magnetic field	High, standard clinical imaging
US imaging	Moderate, ~100–500 μm	High, ~30–100 Hz	Moderate, tissue attenuation and bone occlusion	None	High, immune to magnetic field	High, integrated with robotic tracking
MRI	Moderate, ~200–800 μm	Low, ~0.5–2 Hz	Deep, full- body penetration	None	Moderate, severe susceptibility artifacts from magnetic material	Moderate, limited by temporal latency and magnetic artifact
OA imaging	High, ~100–200 μm	High, ~10–100 Hz	Shallow, ~10 mm	None	High, immune to magnetic field	High, but restricted to superficial targets
SWIR imaging	High, ~50–150 μm	High, ~30–150 Hz	Shallow, ~11 mm	None	High, immune to magnetic field	High, but restricted to superficial targets
Endoscopic imaging	Ultrahigh, ~10–100 μm	High, ~30–60 Hz	None, only cavity surfaces	None	High, immune to magnetic field	High, but requires optical visibility
EMT	High, ~0.2–2 mm, ~0.2°–2°	High, ~40–175 Hz	Deep, ~300–400 mm	None	Moderate, sensor saturation and field distortion	Moderate, need mitigation strategy
Internal magnetic sensor	Moderate, ~0.35–3.5 mm, ~1.5°–5.0°	High, ~10–100 Hz	Moderate, ~80–200 mm	None	Moderate, the same magnetic source for actuation and sensing	High, specially developed
External magnetic sensor	Moderate, ~0.95–7 mm, ~1°–11°	Moderate, ~0.3–2.5 Hz	Shallow, ~40 mm	None	Moderate, low sensing field and strong actuation field	Moderate, need mitigation strategy

MACRs, magnetically actuated continuum robots; MRI, magnetic resonance imaging; OA, optoacoustic; SWIR, shortwave infrared; EMT, electromagnetic tracking; US, ultrasound

### Medical imaging- based methods

X- ray fluoroscopy remains the clinical gold standard for endovascular interventions, providing high- resolution real- time tracking for MACRs. Based on the inherent radiopacity of the robot body or integrated radiopaque markers, these systems facilitate manual identification or automated segmentation of the robot within complex vascular networks (Fig. 4A) [8,35,57,80,81,83,86,95]. In addition to conventional fluoroscopy that remains 2D planar projections, the 3D configuration within intricate anatomical structures can be reconstructed using biplane systems or advanced 2D- to- 3D registration algorithms [98]. However, the main constraint remains the cumulative dose of ionizing radiation for both patients and medical personnel.

**Fig. 4.**
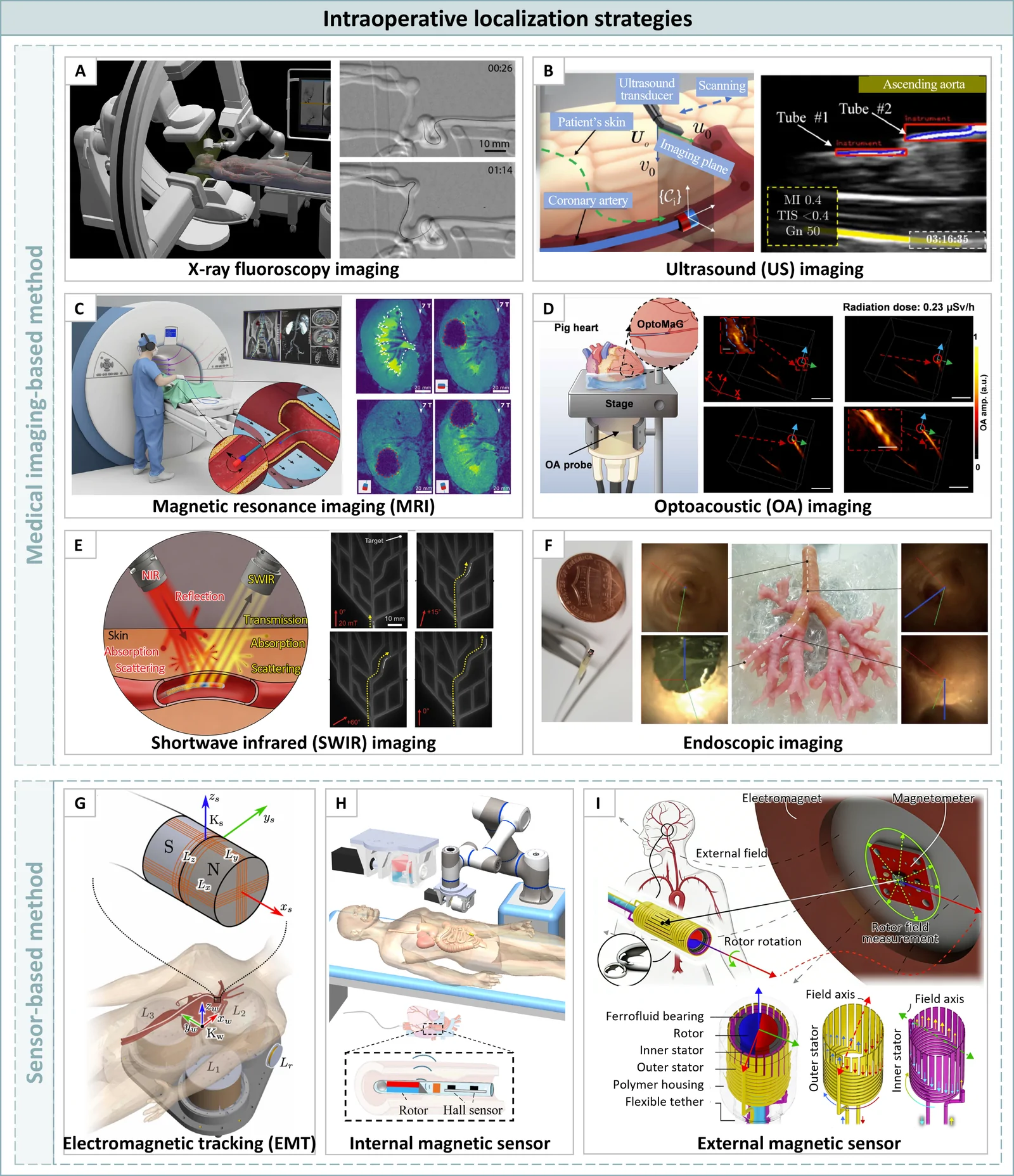
Representative intraoperative localization modalities. (A to F) Medical imaging- based methods: (A) X- ray fluoroscopy- guided neurovascular interventions. Reproduced from Ref. [35]. Copyright 2022, AAAS. (B) Ultrasound (US) imaging- guided coronary interventions. Reproduced from Ref. [77]. Copyright 2024, IEEE. (C) Magnetic resonance imaging (MRI)- guided steering and imaging of a magnetic guidewire. Reproduced from Ref. [34]. Copyright 2023, AAAS. (D) optoacoustic (OA) imaging- guided therapy using an optoacoustically augmented magnetic guidewire. Reproduced from Ref. [6]. Copyright 2026, AAAS. (E) Shortwave infrared (SWIR) imaging- guided peripheral vascular interventions. Reproduced from Ref. [101]. Copyright 2025, Wiley. (F) Endoscopic imaging- assisted control in a lung phantom. Reproduced from Ref. [96]. Copyright 2018, World Scientific. (G to I) Sensor- based methods: (G) Combined electromagnetic actuation and localization based on orthogonal pulse- width modulation. Reproduced from Ref. [103]. Copyright 2025, IEEE. (H) Simultaneous magnetic actuation and localization based on a permanent magnet system. Reproduced from Refs. [107,119]. Copyright 2026, IEEE. (I) Magnetic actuation and localization of locking synchronous motors. Reproduced from Ref. [111]. Copyright 2025, Nature.

US imaging offers a cost- effective, portable, and nonionizing alternative for intraoperative localization. Its high temporal resolution enables real- time monitoring. Researchers have demonstrated various tasks using robotic manipulators or dedicated mechanisms to drive the transducer for continuous tracking (Fig. 4B) [27,46,54,77,78,83,85,99,100]. Despite these advantages, US images are susceptible to speckle noise and acoustic shadowing, which may obscure the submillimetric robot body. Furthermore, its efficacy is limited by penetration depth in the presence of bone or air, and image quality remains highly operator- dependent.

MRI offers good soft- tissue contrast without ionizing radiation, enabling clear differentiation of intricate anatomical structures such as the brain, spinal cord, and vasculature. For MACRs, the primary challenge arises from the compatibility between the robot’s magnetic element and the strong static magnetic field of the MRI system. Consequently, MACRs for MRI environments necessitate specialized materials and structural designs. Although magnetic components can introduce image artifacts, they simultaneously present opportunities for integrated actuation. Numerous studies have explored utilizing the MRI scanner for simultaneous actuation and imaging (Fig. 4C) [33,34,37–39,41,60]. Nevertheless, technical hurdles such as low temporal resolution, severe image artifacts, and complex system integration must be addressed for clinical adoption.

OA imaging is a hybrid radiation- free modality that integrates the high contrast of optical imaging with deep- tissue penetration of US imaging. The primary technical barrier to its clinical translation is the limited penetration depth caused by optical scattering and ultrasonic attenuation. To mitigate this, a recent study introduced an optoacoustically augmented MACR integrated with functional materials, such as ZnS:Cu and FePt nanoparticles. These materials generate robust ultrasonic signals when excited by nonionizing short- pulsed lasers, successfully extending the effective tracking depth to approximately 10 mm (Fig. 4D) [6].

Shortwave infrared (SWIR) imaging has emerged as a novel nonionizing modality, offering spectral tunability, high photostability, and an improved signal- to- background ratio. Researchers have investigated millimetric robots fabricated from quantum dot composites, such as HgCdSe/HgS multishell structures, which emit SWIR signals when exposed to near- infrared light. This approach achieves a tracking depth of approximately 11 mm, demonstrating the feasibility of guiding peripheral vascular interventions (Fig. 4E) [101].

Endoscopic imaging utilizes miniature complementary metal-oxide- semiconductor cameras integrated at the distal tip to provide real- time visual feedback for intraoperative localization. For instance, 5- DoF absolute pose estimation is achieved by combining the alignment of the MACR’s lumen center in endoscopic views across multiple magnetic states with the minimization of torque balance residuals [102]. In addition to global localization, the magnetic field input required to reach a target view can be determined through real- time estimation of the robot’s differential kinematics (Fig. 4F) [96]. Nevertheless, this technology is highly dependent on the intraluminal optical environment and mucosal surface texture, and it may encounter challenges in featureless anatomical cavities, where robust visual tracking is difficult.

### Sensor- based methods

In electromagnetic tracking, the 5- or 6- DoF pose of a robot is determined by sensing voltages induced in its tip- mounted microcoils by a spatially varying magnetic field. Due to its capability for real- time localization without line- of- sight constraints, electromagnetic tracking has become one of the standard technologies for interventional navigation. Commercial systems, such as the Aurora system from Northern Digital Inc. (Waterloo, Canada) and the AxiEM system from Medtronic (Louisville, USA), have been widely adopted in both research and clinical applications. When applied to MACRs, the primary challenge is severe electromagnetic interference between the high- intensity actuation magnetic field and the low- intensity time- varying tracking field. Mitigation strategies include time- division multiplexing to interleave actuation and localization phases, frequency decoupling to separate low- frequency actuation fields from high- frequency tracking signals, and model- based compensation to predict and mitigate actuation- induced disturbances (Fig. 4G) [103,104].

Using internal magnetic sensors is another option. Miniaturized magnetic field sensors, including Hall- effect and magnetoresistive variants, can be integrated into the distal tip of the robot to measure the local magnetic flux density vector generated by the MFGPs. The rapid development of ultraminiaturized sensors has enabled such integration, as exemplified by the AK09973D (1.18 mm × 0.78 mm × 0.55 mm) and AK09940 (0.8 mm × 0.8 mm × 0.4 mm) from Asahi Kasei Microdevices (Tokyo, Japan). Given that the spatial distribution of the actuation field is analytically modeled or precalibrated, these real- time measurements provide position- dependent magnetic field signatures. By solving the magnetic field inversion problem or matching with precomputed field maps, the robot pose can be estimated in real time (Fig. 4H) [105–107]. Furthermore, this approach can be augmented with inertial measurement units to enhance tracking accuracy or provide additional DoFs [108,109].

External magnetic sensors provide an alternative tracking strategy, in which sensors arranged outside the patient’s body measure the magnetic field generated by the robot’s internal magnet. The spatial pose is estimated by solving the magnetic field inversion problem, which requires robust algorithms to separate the weak localization signal from the strong actuation magnetic field. Recent studies have demonstrated localization using a single mobile sensor or stationary sensor arrays (Fig. 4I) [110,111]. This approach eliminates the need for complex distal sensor integration; however, the achievable localization range and accuracy are constrained by the limited size of the internal magnet considering the rapid spatial decay of the magnetic field. To address these limitations, a recent study proposed a portable and flexible intermediary sensing patch that enables a dual- stage localization framework. Unlike conventional rigid arrays, the proposed design adapts to the body contours, improving localization accuracy and robustness [112].

## Control Strategies

Autonomy in MACRs is driven by 2 core factors: environmental sensing reliability and algorithmic complexity. Early- stage studies relied on manual or heuristic control, where operators needed to manually adjust field sources (e.g., the pose of permanent magnets and the currents in electromagnetic coils) through trial and error. With the maturation of modern MFGPs, such primitive approaches have rarely been adopted. By integrating established autonomy frameworks in medical robotics with current technological developments of MACRs, 4 levels of control are defined [1,113]. It should be noted that while general medical robotics typically adopts a 6- level scale (from Level 0 to Level 5), this review uses a truncated 4- level framework. Level 0 is excluded as it is not intuitive and rarely used now, and Level 5 is omitted because full automation remains clinically, ethically, and legally unfeasible in the foreseeable future.

• Level 1 - Robotic assistance: At this level, the operator directly modulates the magnetic field (e.g., field intensities and directions, as well as gradient intensities and directions) according to the actual situation. The system autonomously handles low- level hardware control (e.g., coordinated positioning of robotic manipulators and precise current allocation of multiple electromagnetic coils). This stage achieves hardware transparency, allowing the operator to focus on high- level control of the magnetic environment and task execution [4,5,7–10,33,35,64–66,98].
• Level 2 - Task autonomy: At this level, the control focus shifts from field space to robot space. The operator directly decides the desired pose of the robot (e.g., distal tip position and orientation) according to the actual situation. The system then solves the magnetic field inversion problem to generate the magnetic field required to maintain that pose. A feature of this level is the implementation of closed- loop control, as the compliant nature of MACRs makes them sensitive to environmental interactions. This stage achieves physics transparency, in which real- time localization feedback is incorporated to bridge the gap between model predictions and actual system behavior [11,12,26,27,34,36,46,48,55,59–62,67,69,70,73,81,92,94,95,97,106,107].
• Level 3 - Conditional autonomy: At this level, the system takes over task- level planning (e.g., path planning and trajectory following). The operator transitions from a continuous hands- on controller to a high- level strategic supervisor. The system handles routine navigation, while the operator remains in the loop and ready to intervene when complex anatomical conditions or critical procedural steps arise. This stage streamlines clinical workflows and prevents operator fatigue during prolonged procedures [54,57,74,75,77,78,82,85,96,99,100,104,114].
• Level 4 - High autonomy: At this level, the system is capable of managing the entire clinical workflow (e.g., environment recognition, multimodal scene understanding, strategy generation, and task execution). The robotic system operates in various clinical scenarios with a high degree of autonomy and minimal human intervention. The operator provides ethical and safety oversight, while the system autonomously handles the dynamic and unstructured in vivo environment. This stage relieves the operator of continuous manual input, requiring advanced perception and control frameworks that mirror the sophisticated situational awareness and risk judgment of expert clinicians [115].

Most reported MACR systems currently operate at Levels 1 to 3. The technical challenges associated with high- level autonomy are shared among medical robotic systems, whereas those related to low- level autonomy are closely tied to the unique actuation physics of MACRs. Several challenges arising from magnetic actuation should be addressed during control. First, the distributed nature of magnetic loads along the flexible robot body leads to highly coupled continuum deformation, complicating accurate kinematic and dynamic modeling as well as real- time control. Second, the dynamic response of MACRs is constrained by the bandwidth of magnetic actuation systems and the viscoelastic properties of compliant structures, often resulting in delayed or attenuated motion. Third, strong magnetic actuation can induce structural instabilities, such as buckling, torsional deformation, or loss of shape controllability, thus degrading steering accuracy and operational safety. Finally, accurate force control remains challenging because the tip force is difficult to measure directly in clinical settings and is influenced by robot deformation and environmental contacts. Therefore, the realization of higher levels of autonomy in MACRs requires not only overcoming these low- level physical challenges but also developing planning and control frameworks tailored to magnetic actuation.

## Potential Applications

The clinical utility of MACRs is rooted in their adaptability to diverse physiological environments. Given that organ geometry and tissue mechanics vary substantially, successful deployment requires a task- specific design. As delineated in the taxonomy in Fig. 5, this section reviews MACR applications in major body systems, highlighting recent advances and potential directions to address clinical bottlenecks. The translational stages described in this section represent the overall maturity of MACR applications within each anatomical category, based on the available evidence for the reviewed devices. Platform- level maturity and the clinical use of conventional magnetic instruments are identified separately where applicable.

**Fig. 5.**
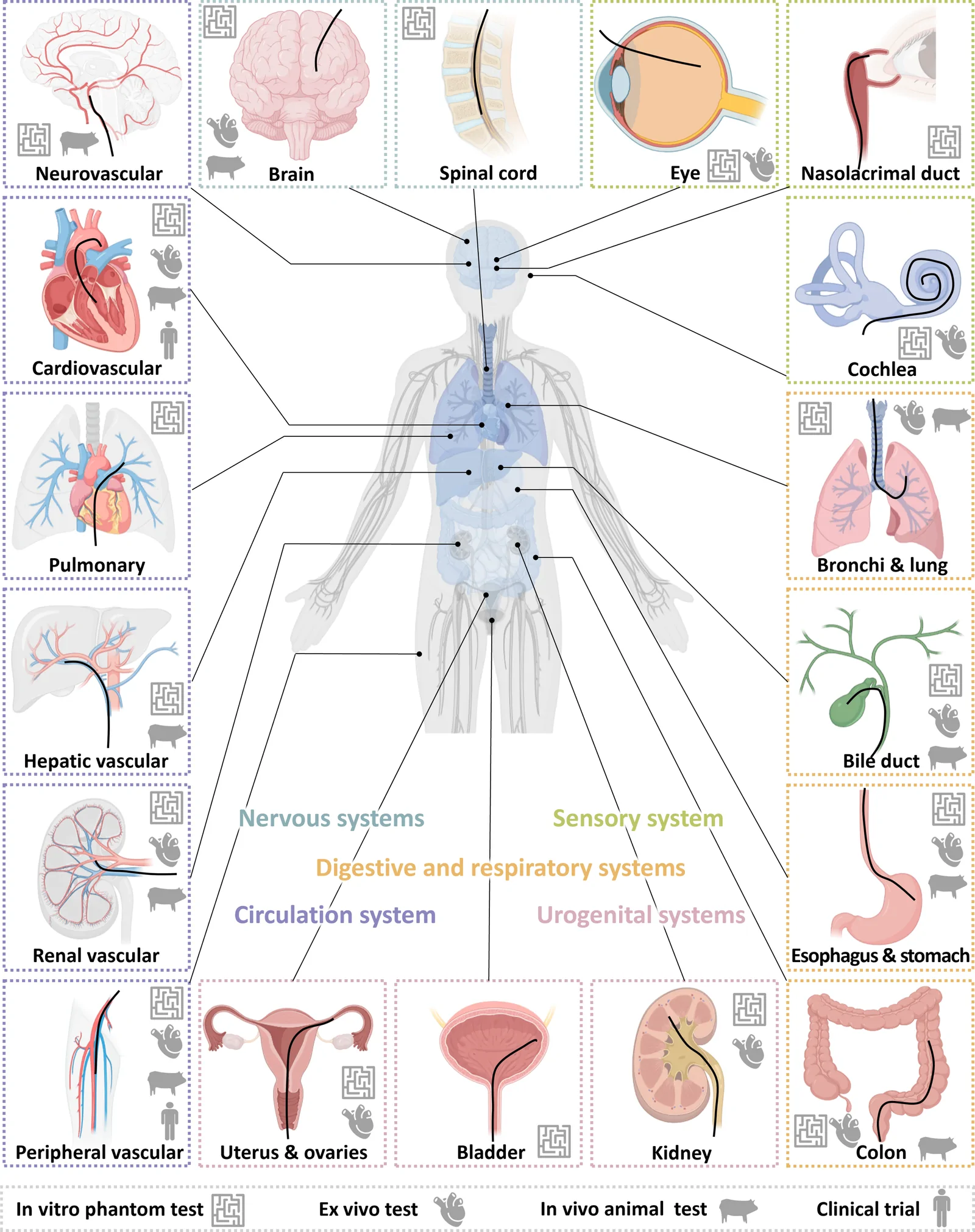
Summary of the potential applications of magnetically actuated continuum robots by anatomical systems, labeled with current translational stage. Adapted and redrawn from Ref. [15]. Copyright 2023, Wiley. Created with BioRender.

### Circulatory system

The circulatory system consists of a large vascular network, which enables the hemodynamic transport of nutrients, oxygen, and hormones throughout the body. For MACR- based interventions, this system primarily includes the cardiovascular system (e.g., heart and coronary arteries), the neurovascular system (e.g., cerebral vessels), the peripheral vasculature (e.g., limb arteries), the visceral vasculature (e.g., renal arteries and hepatic arteries), and the pulmonary circulation. Among these vascular applications, cardiovascular and peripheral vascular interventions are supported by the most mature magnetic navigation platforms, as demonstrated by the commercial deployment and clinical evaluation of magnetic navigation systems and conventional magnetically guided catheters. In contrast, the newly developed MACRs reviewed herein remain at the preclinical stage, with validation primarily limited to in vitro phantoms, ex vivo organs, and in vivo animal models.

The clinical appeal of MACRs lies in their enhanced steerability, which enables their deployment as active endovascular instruments (e.g., guidewires and catheters) to negotiate tortuous bifurcations and stenotic lesions [4,9]. A primary application is targeted delivery. For example, MACRs can precisely route contrast agents for high- fidelity angiography, as well as administer thrombolytic and chemotherapeutic agents directly to the site, maximizing local impact while avoiding systemic toxicity to the patient [64]. Furthermore, MACRs can improve the precision of stent placement in small- diameter vessels and the deployment of embolic agents (e.g., coils and liquid adhesives) to treat aneurysms and arteriovenous malformations [35]. Beyond delivery, MACRs excel in complex interventions that require active mechanical interaction and stable positioning. For example, they can provide the stable contact force necessary for effective radiofrequency ablation to treat cardiac arrhythmias [73,74]. Specialized robots can perform mechanical thrombectomy and restore blood flow in cases of acute ischemic stroke or chronic total occlusions [81,83,85]. MACRs also extend to advanced diagnostics. Their precise force control enables safer intravascular or endomyocardial biopsies with reduced risk of tissue perforation during sampling [71,80,89]. By mitigating frictional resistance and force transmission losses inherent in conventional instruments, these robots also facilitate intravascular imaging when integrated with high- resolution probes such as intravascular US or optical coherence tomography, allowing in situ evaluation of stent placement and vascular morphology [26,97].

### Nervous system

The nervous system coordinates physiological functions by transmitting bioelectric signals through distinct anatomical regions. For MACR- based interventions, this system mainly includes the central nervous system (e.g., brain and spinal cord) and the peripheral nervous system (e.g., cranial nerves and spinal nerves). Given the fragility of neural structures, these applications are being verified through phantom experiments and in vivo animal tests to evaluate safety and navigation efficiency.

Due to the delicate and highly organized nature of neural tissues, the primary appeal of MACRs in this domain lies in their capacity for steerable and high- precision interstitial navigation along nonlinear trajectories. This capability enables access to anatomically confined regions that remain unreachable for conventional rigid needles [50,51]. Upon reaching intracranial or intraspinal targets, these robots facilitate targeted drug delivery and biopsy, enabling localized administration of neurotrophic agents or chemotherapeutics to minimize systemic exposure, as well as high- fidelity tissue sampling for precise in situ diagnostics. Building on this capability, MACRs offer a safer alternative to electrode implantation and neuromodulation. This is important for applications such as deep brain stimulation or spinal cord stimulation, where distal dexterity ensures optimal electrode- neural interface coupling, substantially reducing the risk of accidental hemorrhage during insertion [116]. Furthermore, MACRs enable microscale surgical manipulation within deep- seated lesions, effectively preserving functional neural circuits while broadening the scope of minimally invasive neurosurgical procedures [90].

### Digestive and respiratory systems

The digestive and respiratory systems share common anatomical access routes and consist of extensive, highly branched luminal structures. The digestive system is responsible for ingestion, digestion, and absorption of nutrients, encompassing the esophagus, stomach, small and large intestines, and bile ducts. The respiratory system enables gas exchange and pulmonary ventilation, including the trachea, bronchi, and peripheral lung regions. Currently, these applications are being validated through phantom experiments, ex vivo organ and cadaveric trials, and in vivo animal tests. These applications have strong translational potential because steerable flexible endoscopes (e.g., gastroscopes, colonoscopes, and bronchoscopes) are already widely used in standard clinical workflows. Furthermore, the established success of magnetic capsule endoscopy as a routine clinical modality underscores the practical effectiveness of magnetic actuation in endoluminal environments.

Current endoscopic technologies are often hampered by a reach problem, specifically the inability to navigate acute flexures or access distal lesions. MACRs address these limitations through their miniaturized footprint and distal dexterity, with examples including negotiating distant generations of the bronchial tree or sharp flexures of the bile duct [8,11,70]. Upon reaching the target, the robots can perform high- resolution optical and nonoptical imaging modalities for early- stage tumor detection, as well as precise biopsy for in situ assessment of mucosal abnormalities [96]. Beyond diagnostics, the ability to deliver drugs or inject therapeutic agents directly into suspicious nodules increases therapeutic efficacy while sparing surrounding healthy tissues [46]. These robots also support a broad spectrum of endoluminal procedures, including polyp removal, stone extraction, foreign- body retrieval, stent placement, and localized ablation of mucosal malignancies [66,74,75,108].

### Urogenital system

The urogenital system is responsible for waste excretion and reproduction. These narrow, winding channels provide a challenging yet promising workspace for MACRs. In the urinary tract, potential applications include the kidneys, ureters, bladder, and prostate. In the reproductive system, these applications extend to the ovaries, uterus, and fallopian tubes. Existing applications are being tested mainly in phantom experiments and ex vivo models. Unlike the extensive and intricate networks found in the vasculature, the relatively shorter and less branched anatomy presents a less urgent clinical demand for remote magnetic guidance, which may explain the limited studies in this specific domain. Nevertheless, they have received more attention in recent years and have great translational potential, with the aim of further improving the precision and flexibility of existing clinical instruments.

In urology, the defining challenge involves navigating the narrow and winding ureter to access the renal pelvis without causing mucosal trauma. The superior intraluminal maneuverability of MACRs provides the dexterity required for retrograde intrarenal surgery, where conventional passive endoscopes are constrained by acute access angles [31]. Once positioned at the target site, these robots facilitate in situ biopsy of suspicious urothelial lesions and localized ablation of tumors within the bladder or prostate [94]. This distal dexterity is transformative for calculus management. MACRs can precisely guide interventional tools (e.g., laser fibers and retrieval microbaskets) into anatomically hostile renal calyces to facilitate effective lithotripsy. Furthermore, these robots enable targeted drug delivery, such as localized injection of chemotherapeutic agents directly into the bladder wall or prostate tissue while preserving surrounding healthy tissues. In the realm of reproductive medicine, submillimetric MACRs unlock unique capabilities for high- precision interventions in the female reproductive tract. Their ability to navigate the delicate environment of the fallopian tubes and perform micromanipulation of oocytes represents a leap for assisted reproductive technologies, offering a potential solution for treating tubal factor infertility [62,80,117].

### Sensory system

Sensory systems comprise specialized organs that transduce physical or chemical stimuli into neural signals, allowing environmental perception and spatial orientation. In the context of MACR- based interventions, this domain spans the visual system (e.g., intraocular space and subretinal layers), the auditory and vestibular system (e.g., cochlea and semicircular canals), the olfactory system (e.g., nasal cavity and skull base), and their associated anatomical conduits (e.g., nasolacrimal and Eustachian ducts). As a relatively new field, research in this category is still in its infancy and is primarily concentrated on phantom experiments as well as ex vivo organ and cadaveric evaluations. These applications demonstrate strong potential for clinical translation, owing to their potential for miniaturization and active steering.

Operation in sensory organs demands a level of precision that pushes the limits of human dexterity, particularly within confined, fragile, and often inaccessible anatomical spaces. MACRs offer a unique capacity for precise and atraumatic navigation through microscale channels. A representative application is cochlear implantation, where MACRs can be programmed to conform to the spiral geometry of the cochlea. This approach reduces the insertion forces and the risk of trauma to the delicate basilar membrane, thereby supporting the preservation of residual hearing [52]. Beyond the inner ear, MACRs are well suited for ophthalmic surgery, such as retinal vein cannulation and drug delivery to the subretinal space [53,69]. Moreover, the integration of miniaturized endoscopic modules into MACRs enables real- time intraluminal visualization and interventions for obstructive conditions in narrow sensory- related ducts. For example, they can traverse the nasolacrimal duct to resolve chronic blockages without the need for external facial incisions and navigate the Eustachian tube through the nasopharynx to restore middle- ear pressure [118].

## Conclusion and Outlook

The evolution of MACRs has been documented in Fig. 6. Although the field has witnessed remarkable progress in fundamental principles, robot designs, intraoperative localization, and control strategies in various clinical applications, a translational gap is still observed between laboratory prototypes and standardized clinical implementation. For the potential of MACRs in MIS to be fully realized, it is important to analyze existing translational bottlenecks and future development directions.

**Fig. 6.**
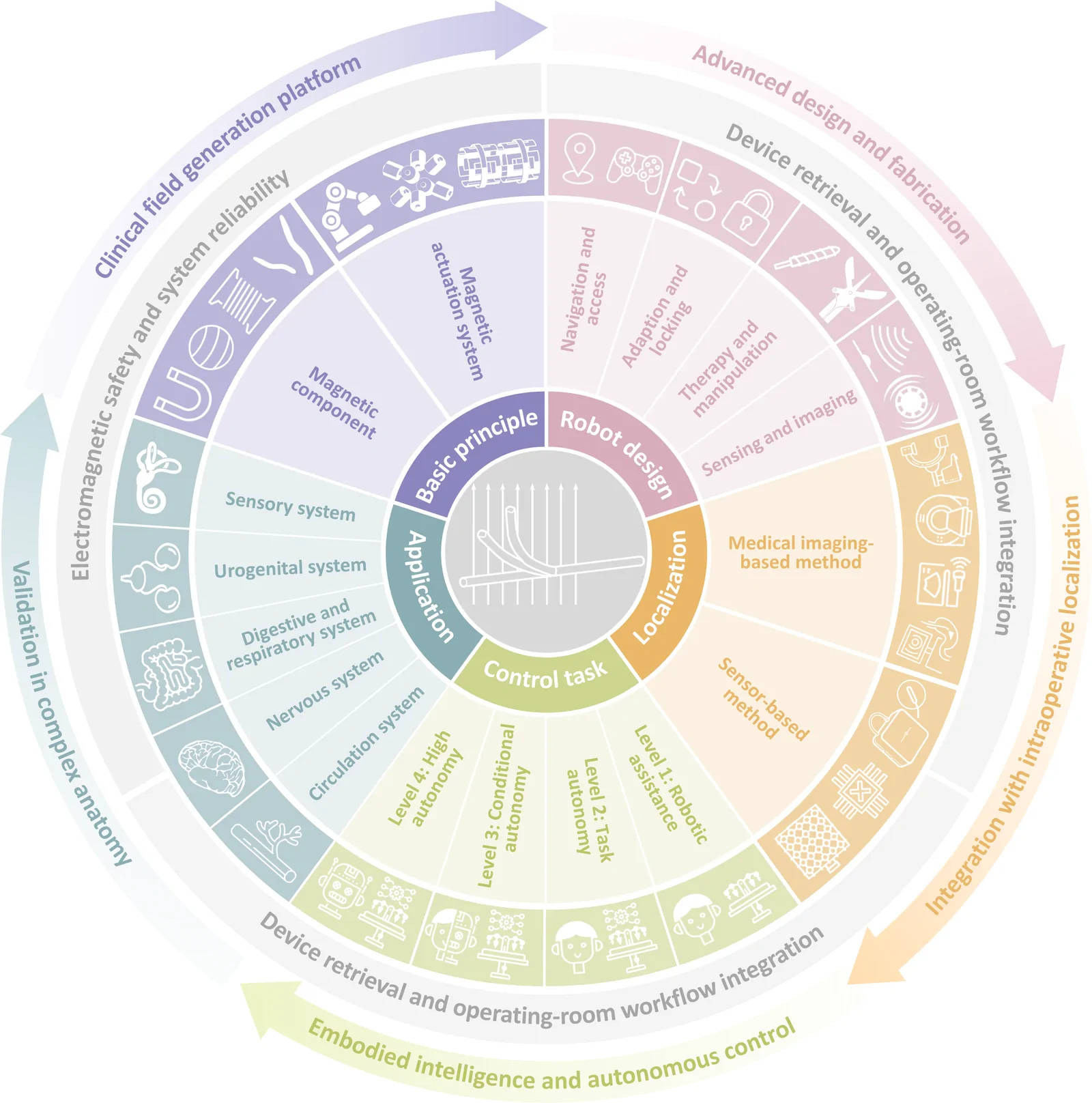
Schematic illustration of magnetically actuated continuum robots from engineering to clinical translation.

### Barriers to translation

Unlike in vitro phantom and in vivo animal studies, clinical deployment demands strict sterilization protocols, regulated medical environments, and maximum safety. Clinical translation entails combined engineering, biological, and operational challenges that are absent in laboratory settings.

• Electromagnetic safety and system reliability: Although dedicated standards specifically for surgical magnetic actuation have yet to be established, clinical MFGPs are mandated to ensure that their generated magnetic fields do not compromise the functionality of surrounding precision infrastructure or distort critical clinical instrumentation, such as C- arm imaging systems, mechanical ventilators, and vital signs monitors. Currently, system evaluation can be conducted in alignment with relevant regulatory frameworks, such as general requirements for medical electromagnetic compatibility (IEC 60601-1- 2), particular requirements for magnetic resonance equipment (IEC 60601- 2- 33), and particular requirements for robotically assisted surgical devices (IEC 80601- 2- 77). Furthermore, the integration of fail- safe architectures remains a prerequisite at the system level. For movable systems, the platform must feature immediate self- locking in the event of power loss to prevent gravity- induced structural sagging and enforce strict collision avoidance during robotic manipulation. For permanent magnet- based systems, because the magnetic field is always- on, rapid mechanical shielding serves as a viable option to isolate field sources within milliseconds during critical failures. For current- dependent systems, appropriate architectures are required to safely dissipate inductive energy and suppress transient thermal spikes once sudden power loss or cooling failure occurs.
• Material biocompatibility and clinical sterilization: Some materials used in existing MACR studies lack sufficient biocompatibility and must be replaced with alternatives that strictly comply with medical- grade standards. Their biological evaluation can refer to established frameworks (e.g., the ISO 10993 series) and specific product regulations governing the corresponding interventional devices. Furthermore, guaranteeing biological and structural integrity remains paramount. For magnetic composites, because embedded magnetic particles introduce potential cytotoxicity and chemical leaching risks, the encapsulation layers must maintain a hermetic seal under repetitive bending to prevent particle exposure. For permanent magnets and ferromagnetic components, manufacturing processes must eliminate the risk of distal joint detachment under dynamic physiological loads. For MACRs that use microcoils, the structural insulation must reliably withstand elevated temperatures and prevent electrical leakage into surrounding tissues. In addition, validating disinfection and sterilization processes is imperative within a regulated medical quality management framework (e.g., ISO 13485) to ensure that they do not compromise material properties or structural functionalities. Conventional high- temperature autoclaving can alter the thermomechanical properties of certain polymers or coating layers and trigger the thermal demagnetization of embedded magnetic components. Alternatively, low- temperature sterilization (e.g., ethylene oxide) could be a feasible pathway.
• Device retrieval and operating- room workflow integration: Integrating MACRs into the operating room necessitates compatibility with surgical workflows to maintain procedural efficiency. Unlike laboratory settings, the operating room is space- constrained, demanding that the physical footprint of MFGPs does not obstruct the access of medical personnel or impede the clearance required for intraoperative imaging systems. Furthermore, a dedicated human–machine safety override module is essential to guarantee that clinician commands unconditionally supersede robotic regulation during intraoperative emergencies. For master- slave architectures, the system must support instantaneous control- loop severance, enabling immediate manual takeover and transition of the device from active robotic control to passive handling. To complement this control- loop breach, the physical actuation or delivery interface must incorporate passive decoupling mechanisms to allow manual withdrawal from tortuous anatomical pathways. Any advanced functional designs, such as active variable- stiffness tuning, microdrilling mechanisms, or detachable wireless modules, must prioritize patient safety. Their operational stability and fail-safe performance should be thoroughly validated within dynamic in vivo environments to eliminate risks of tissue perforation, vascular damage, or device loss during procedures.

### Future directions

Based on current translational barriers and research processes, future research should focus on the following 5 aspects:

• Clinical MFGP: Although substantial progress has been achieved in laboratory settings, scaling MFGPs for clinical deployment requires optimizing their physical footprint, magnetic power density, and intraoperative workspace accessibility. While satisfying the foundational prerequisites for electromagnetic safety and systemic reliability, future developments should focus on structural topological refinement and advanced core configurations to realize patient- centric, space- efficient, and easily deployable platforms. Two engineering pathways can be identified. For permanent magnet- based systems, optimization is directed toward multiaxis robotic manipulation mechanisms to enhance spatial agility and dynamic responsiveness. For electromagnetic architectures, research focuses on optimizing coil distributions, elevating magnetic power capacity, and enhancing cooling efficiency to collectively achieve high- density magnetic field generation.
• Advanced design and fabrication: The operational capacity of MACRs is dictated by the interplay of material mechanics, structural configurations, and microfabrication methodologies. Current development is rapidly transitioning from conventional catheters or guidewires equipped with discrete permanent magnets toward integrated multifunctional composite architectures. To facilitate this technological evolution, research efforts should focus on 2 primary frontiers, both strictly predicated on the utilization of medical- grade substrates that satisfy strict biocompatibility and sterilization tolerances. First, submillimetric robotic bodies should be fabricated to balance compliance and resistance to buckling. Second, multifunctional integration should be achieved by embedding smart materials and responsive structures that support programmable shape- morphing, on- demand stiffness control, localized therapy, and multimodal sensing or imaging. Through these advances, MACRs are expected to be transformed from basic delivery instruments into intelligent systems capable of executing complex interventional procedures.
• Integration with intraoperative localization: Achieving high- precision closed- loop control of MACRs requires the systematic integration of multimodal localization frameworks. Initial efforts should focus on establishing robust spatial coregistration between the coordinate frames of the magnetic actuation workspace and intraoperative clinical imaging, and enhancing the radiopacity and visibility of miniaturized robots under x- ray fluoroscopes. Furthermore, navigation strategies are expanding into nonionizing imaging modalities, including US, MRI, OA, SWIR, and endoscopic imaging, but their working scenario and procedural integration need further validation. In addition, the integration of clinical imaging modalities with soft sensing technologies, such as those utilizing electromagnetic induction or Hall- effect sensing, is increasingly investigated, in which intrinsic perception serves as a complement to external navigation, but the accuracy and interference issues need further consideration. Through such combined localization, exposure to ionizing radiation can be reduced, and physiological artifacts such as respiration and heartbeat can be effectively compensated. Therefore, robust tracking and registration are enabled within complex and dynamic in vivo environments.
• Embodied intelligence and autonomous control: Driven by breakthroughs in modern control theory and artificial intelligence, MACRs are evolving from conventional manual teleoperation toward embodied intelligence. Future control architectures are poised to achieve higher levels of surgical autonomy by fusing preoperative anatomical maps with multimodal intraoperative imaging and sensory feedback. This cognitive evolution supports the autonomous execution of procedural workflows, including optimized global path planning through branched anatomical trees, active obstacle avoidance within dynamic luminal boundaries, and task- specific interventional maneuvers. Furthermore, establishing robust shared control frameworks between surgeons and intelligent robotic systems optimizes the human- in- the- loop dynamic. This collaborative synergy can mitigate the cognitive burden of the clinician while maximizing surgical precision, safety, and overall procedural outcomes.
• Validation in complex anatomy: Transitioning MACRs toward clinical readiness requires rigorous evaluation within high- fidelity, nonidealized testing environments. Although preliminary in vivo demonstrations have been reported, systematic and scaled validation pathways remain scarce. Future evaluation must progressively transition from static geometric phantoms toward dynamic in vivo large- animal models, and ultimately comprehensive clinical trials. This shift is critical for assessing device performance within unstructured and dynamic physiological environments, such as highly narrow and tortuous neurovascular and bronchial branches, high- velocity hemodynamic fields in cardiology, and complex luminal environments characterized by tissue friction and biological obstructions. Within these translational contexts, validation metrics should prioritize the evaluation of active navigability through real anatomical networks, the stability of magnetic alignment in deep- seated lumens, and the long- term biological safety of devices.
